# Does Walnut Supplementation Have Favourable Effect Apolipoprotein A, B and Blood Pressure? A Systematic Review, Meta‐Analysis and Meta‐Evidence of Randomised Clinical Trials

**DOI:** 10.1002/edm2.70171

**Published:** 2026-03-20

**Authors:** Vali Musazadeh, Mahsa Mahmoudinezhad, Seyed Mohammad Hosseini‐Roknabadi, Maryam Falahatzadeh, Farzad Shidfar

**Affiliations:** ^1^ Student Research Committee, School of Public Health Iran University of Medical Sciences Tehran Iran; ^2^ Department of Nutrition, School of Public Health Iran University of Medical Sciences Tehran Iran; ^3^ Student Research Committee Urmia University of Medical Sciences Urmia Iran; ^4^ Department of Nutrition, School of Medicine Urmia University of Medical Sciences Urmia Iran; ^5^ Department of Pharmacy Shiraz University of Medical Sciences Shiraz Iran

**Keywords:** apolipoprotein, blood pressure, meta‐analysis, walnut

## Abstract

**Backgrounds:**

Many randomised controlled trials (RCTs) have revealed the benefits of walnut on apolipoproteins and blood pressure, but the results are inconclusive. This meta‐analysis of RCTs aimed to assess the effects of walnut on Apolipoprotein B (ApoB), apolipoprotein A1 (ApoA1) and blood pressure.

**Methods:**

A systematic review of PubMed, Scopus, Web of Science, Cochrane and Embase databases was conducted, and the search time frame was from the establishment of the database up to January of 2025. A random effects model was applied to estimate weighted mean differences (WMDs) and 95% confidence intervals (CIs).

**Results:**

Twenty‐five RCTs comprising 26 intervention arms with 2155 patients were included. Walnut significantly decreased ApoB (WMD = −0.06; 95% CI: −0.10, −0.01, *p* = 0.002), but did not affect ApoA1 (WMD = −0.50; 95% CI: −1.34, 0.33, *p* = 0.249), systolic blood pressure (SBP) (WMD = −1.20; 95% CI: −4.02, 1.61, *p* = 0.401) and diastolic blood pressure (DBP) (WMD = −0.44; 95% CI: −2.55, 1.67, *p* = 0.682).

**Conclusion:**

Walnut intake was associated with reduced ApoB levels, with no significant effects observed on ApoA1, SBP, or DBP. Future research involving large‐scale, international RCTs is essential to validate its therapeutic potential further.

## Graphical abstract

This meta‐analysis of 25 randomised controlled trials demonstrates that walnut consumption significantly lowers apolipoprotein B levels while showing no significant effects on apolipoprotein A1, systolic blood pressure, or diastolic blood pressure.

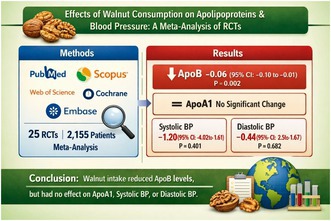

## 1 Introduction

Over the last three decades, a substantial body of evidence has been conducted regarding the management of hypertension (HTN) [1]. HTN has a drastic correlation with age, and this correlation is more evident in Blacks than Whites [2]. It is estimated that more than 1 billion people worldwide suffer from HTN [3]. Both the American Heart Association and the European Society of Hypertension recommend non‐pharmacological interventions such as reducing sodium intake, achieving weight loss and following a diet rich in fruits, vegetables and low‐fat dairy products, as outlined in the DASH (Dietary Approaches to Stop Hypertension) eating plan [2, 4]. Dyslipidemia commonly occurs alongside HTN within the general population [5]. The INTERHEART study demonstrated that the presence of just one risk factor can double or triple the overall risk of cardiovascular diseases. However, when risk factors such as HTN, dyslipidemia, diabetes and smoking occur together, the total risk increases more than 20‐fold [6]. In recent years, complementary and alternative medicine (CAM) has garnered growing attention for its perceived value among individuals seeking proactive approaches to health [7]. Examples include natural products, mind–body medicine (MBM), yoga, traditional Chinese medicine, dietary modifications and numerous other integrative health practices [8]. One of these approaches that has recently gained attention is the dietary inclusion of walnuts.

Walnuts ( *Juglans regia* L.), a key element in Mediterranean and Asian cuisines, are gaining popularity globally across different dietary practices because of their dense nutrient composition and associated health‐promoting properties [9]. In addition, walnuts have maintained a prominent position in the functional food market, supported by extensive evidence of their nutritional and therapeutic benefits. Recently, their lipid composition has garnered considerable scientific interest, as it has been associated with a range of biological functions and health benefits [10]. Walnuts are unique among nuts for their substantial content of alpha‐linolenic acid (ALA), a plant‐derived omega‐3 fatty acid that makes up to 9% of their total weight [11].

To the best of our knowledge, this is the first meta‐analysis which assesses the effects of walnut on apolipoproteins A and B with blood pressure outcomes, comprehensively. There has been a considerable amount of evidence regarding walnuts and its effects on blood pressure or lipid levels. The previous meta‐analysis on this topic was conducted in 2020 [12]. However, since then, new data have emerged with the potential to either modify or confirm the findings of the earlier meta‐analysis. In addition, the current study focuses on apolipoproteins and integrates the blood pressure values which may provide a more comprehensive capture of walnut's effect on metabolic health. Therefore, we decided to perform a systematic review and meta‐analysis of all the available data on this subject.

## 2 Method

The study followed the guidelines of the PRISMA statement (Preferred Reporting Items for Systematic Reviews and Meta‐Analyses) [13]. Furthermore, the study protocol was registered in the International Prospective Register of Systematic Reviews (PROSPERO) with ID: https://www.crd.york.ac.uk/PROSPERO/view/CRD420251009109.

### 2.1 Search Strategy

A comprehensive and systematic search was conducted on multiple databases such as PubMed, Scopus, Cochrane, Embase and Web of Science databases. The systematic search was performed using keywords relevant to this study's topic. More detailed information is available in Table S1. Additionally, no language restrictions were imposed on the search process, and we gathered all the available and relevant data up until January of 2025. Furthermore, we conducted a manual review of the reference lists, along with studies that cited the included trials and relevant review articles.

### 2.2 Study Selection

The PICO criteria for this meta‐analysis were as follows: Population/Patients (P) included adults aged 18 years and older who consumed walnuts; Intervention (I) involved walnut consumption; Comparison (C) was a control or placebo group; and Outcome (O) focused on Apolipoprotein B (ApoB), apolipoprotein A1 (ApoA1), diastolic blood pressure (DBP) and systolic blood pressure (SBP). The results were independently screened by two researchers, and any disagreement was resolved by a third researcher (Kappa = 0.88). We included studies that (a) employed a randomised controlled design with either parallel or crossover design, (b) investigated the effect of walnut consumption on SBP, DBP or ApoB and ApoA1 concentrations, and (c) provided sufficient information to calculate the effect size for both the intervention and control groups. The exclusion criteria for this article were as follows: (a) experimental, (b) observational in design and (c) in vitro and in vivo studies.

### 2.3 Data Extraction

Two independent reviewers (MM and VM) screened and assessed the results of the systematic search across various databases. Initially, the articles were reviewed based on their titles and abstracts, and relevant studies were shortlisted for full‐text review. In cases where there was a disagreement between the reviewers, a third colleague (FSH) acted as an arbitrator to resolve the differences. From the studies that met the eligibility criteria, we extracted the following information, including but not limited to: the first author's name, publication date, study location, number of participants in both the intervention and control groups, walnut dosage, treatment duration, participants' age and gender, as well as pre‐ and post‐intervention data on SBP, DBP, or ApoB and ApoA1.

### 2.4 Methodological Quality and Meta‐Evidence

We will assess the quality of the included studies using the Cochrane Risk of Bias Assessment Tool, including risk of bias assessment for randomization methods, allocation concealment and blind implementation [14]. The overall certainty of the evidence was evaluated using the GRADE (i.e., Grading of Recommendations Assessment, Development and Evaluation) method [15].

### 2.5 Data Synthesis and Statistical Analysis

The statistical analysis of the extracted data was done using STATA software version 16.0 (STATA Corp, College Station, TX, USA). We used the mean difference and standard deviation between baseline and at the end of the study in both intervention and control groups to conduct this meta‐analysis. Moreover, weighted mean differences (WMDs) with 95% confidence intervals were calculated using a random effects model [16]. To determine the changes in standard deviation (SD), the following formula was utilised: SD change = square root ([SD baseline]^2^ + [SD final]^2^ – [2R × SD baseline × SD final]), where R = (SD1^2^ + SD2^2^—SDchange^2^)/(2 × SD1 × SD2). When data were presented as standard errors (SEs), interquartile ranges (IQRs), or 95% confidence intervals (CIs), the mean ± standard deviation (SD) values were calculated. Inter‐study heterogeneity was evaluated using the random‐effects model (employing the DerSimonian‐Laird method), the Cochran Q test and the *I* ^2^ index. Significant heterogeneity between studies was defined as *I* ^2^ values exceeding 50.5% or *p*‐values below 0.1. Sensitivity analysis was carried out to evaluate the influence of individual trials. Moreover, using meta‐regression analysis, we examined the presence of any linear relationship between observed effect size and age, treatment duration and dosage of trials. Additionally, small‐study effects were examined using Egger's and Begg's tests [17, 18]. In order to assess publication bias, a funnel plot was used. In cases of significant publication bias, the ‘trim and fill’ analysis was applied.

## 3 Results

### 3.1 Study Selection

**FIGURE 1:**
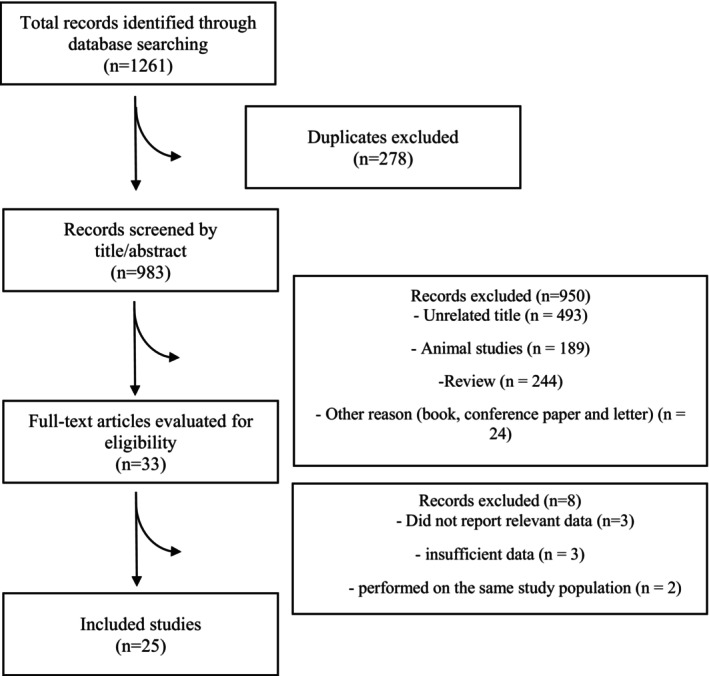
Flow diagram of study selection.

We retrieved 1261 publications from multiple databases through systematic search, out of which 278 were duplicates and subsequently excluded. Following the primary screening process, based on title and abstract evaluation, an additional 950 papers were excluded. The 33 remaining papers underwent a secondary screening process for full‐text evaluation, during which eight articles were excluded by the following reasons: review articles and studies involving co‐treatment (Table S2). At last, 25 articles met our inclusion criteria and were included in the current study (Figure 1). Six studies reported ApoA1, eight studies reported ApoB, twenty studies reported SBP, and eighteen studies reported DBP.

### 3.2 Study Characteristics

In the present meta‐analysis, 25 studies (26 arms) encompassing 2204 individuals were included. These studies were published from 1997 to 2020. Most of the included studies were conducted in the USA [11, 19, 20, 21, 22, 23, 24, 25, 26, 27, 28], four studies in Spain [19, 29, 30, 31], three in Iran [32, 33, 34], two in New Zealand [35, 36], one in South Korea [37], Australia [38], Korea [39], Scotland [40], China [41] and India [42]. Most studies included both male and female participants, whereas four were conducted exclusively among men [26, 35, 40, 42] and three exclusively among women [21, 33, 39]. The mean age of included participants was 46.6 years old. Most of the individuals were treated with walnuts in their diet, while one study used walnut capsules [32] and another used walnut oil [34]. The treatment dose ranged from 15 to 56 g per day and the treatment period varied from 4 to 104 weeks. Baseline characteristics of study participants are provided in the Table 1.

**TABLE 1:** Baseline characteristics of study participants.

Author name	Country	Gender	Mean age (Int, Con)	N (Int, Con)	Health condition	Intervention type (Int)	Dose	Intervention type (Con)	Duration (week)	Main outcome (IN)	Main outcome (CON)
Abdrabalnabi et al. [19]	USA, Spain	F/M	68.24, 67.51	324, 312	MetS	In diet	15% total energy (45 g/day)	Control diet	104	SBP: −1.3 ± 15.18 DBP: −0.08 ± 8.90	SBP: 0.01 ± 14.73 DBP: −0.71 ± 8.96
Sanchis et al. [31]	Spain	F/M	71, 71	13, 13	CKD	In diet	30 g/day	Rich olive oil diet	4	SBP: −4.0 ± 25.75	SBP: −5.0 ± 21.15
Hwang et al. [37]	South Korea	F/M	41.05, 37.91	41, 41	MetS	In diet	45 g/day	Control food: white bread	16	SBP: −3.07 ± 6.69 DBP: −0.07 ± 5.12	SBP: −4.77 ± 6.79 DBP: −3.97 ± 4.95
Fatahi et al. [33]	Iran	F	54.01, 52.9	33, 33	Overweight and obese	Whole walnut	18 g/week walnut	300 g fish/week	12	SBP: −4 ± 4.79 DBP: −2.7 ± 3.92	SBP: −6 ± 4.35 DBP: −3.3 ± 3.99
Rabiei et al. [32]	Iran	F/M	50.5, 49.9	20, 20	High risk CVD type 2 diabetes	Capsule	100 mg *J. regia* leaf extract	Placebo	8	SBP: −5.0 ± 4.14 DBP: −1.8 ± 3.16	SBP: −1.2 ± 4.62 DBP: 2.9 ± 5.86
Ndanuko et al. [38]	Australia	F/M	43.2, 45.2	82, 62	Obese patients with BMI	In diet	30 g/day	Control: dietary advice	12	SBP: −7.0 ± 11.31 DBP: −4.4 ± 7.62	SBP: −7.0 ± 11.24 DBP: −1.2 ± 8.43
Zibaeenezhad et al. [34]	Iran	F/M	55.5, 44	45, 45	T2DM	Walnut oil	15 g/day	No intervention	12	SBP: −0.24 ± 6.36 DBP: −0.2 ± 3.35	SBP: −0.15 ± 7.54 DBP: −4.9 ± 3.09
Rock et al. [25]	USA	F/M	53.3, 52.2	48, 49	Overweight and obese	In diet	15% total energy	Standard reduced energy density diet	12	SBP: −8.0 ± 10.45 DBP: −6.0 ± 8.33	SBP: −6.0 ± 6.32 DBP: −50 ± 3.16
Rock et al. [25]	USA	F/M	53.3, 52.2	46, 49	Overweight and obese	In diet	15% total energy	Standard reduced energy density diet	24	SBP: −6.0 ± 6.28 DBP: −5.0 ± 8.43	SBP: −4.0 ± 8.18
Njike et al. [23]	USA	F/M	56.5, 53.3	56, 56	High risk for diabetes	In diet	392 g/week	Ad libitum diet	24	SBP: −0.46 ± 11.2 DBP: 0.46 ± 6.42	SBP: 2.38 ± 13.33 DBP: 0.6 ± 7.36
Burns‐Whitmore et al. [20]	USA	F/M	38, 38	20, 20	Healthy	In diet	28.4 g/day	Lacto‐ovo‐vegetarians	6	Apo A: 0.02 ± 0.04 Apo B: −0.03 ± 0.08	Apo A: 0 ± 0.05 Apo B: −0.10 ± 0.08
Lee et al. [39]	Korea	F	No	30, 30	MetS	In diet	15 g/day	Control diet	6	SBP: −5.23 ± 8.26	SBP: −4.8 ± 11.1
Wu et al. [11]	USA	F/M	60, 60	24, 28	Healthy	In diet	43 g/day	Western‐diet	8	Apo B: −0.05 ± 0.13 DBP: −4.2 ± 7.41	Apo B: −0.02 ± 0.11 DBP: −4.4 ± 6.73
Olmedilla‐Alonso et al. [29]	Spain	F/M	54.4, 54.4	25, 25	CVD	In diet	19.4 g/day	Control diet	5	SBP: −2.3 ± 20.28 DBP: −2.4 ± 3.31	SBP: −10.8 ± 27.55 DBP: −0.4 ± 17.98
Katz et al. [22]	USA	F/M	54.7, 54.7	22, 18	Overweight and obese	In diet	56 g/day shelled	Control diet	8	SBP: −2.6 ± 11 DBP: −3.6 ± 18.8	SBP: 1.2 ± 10.7 DBP: −0.6 ± 7.7
Kalgaonkar et al. [21]	USA	F	31.2, 31.2	17, 14	PCOS	Whole walnut	36 g/day	Whole almond nut	6	Apo B: −0.08 ± 0.08	Apo B: −0.07 ± 0.12
Din et al. [40]	Scotland	M	23, 23	15, 15	Healthy	In diet	15 g/day	Control diet	4	SBP: 3.0 ± 4.35 DBP: 3.0 ± 3.49	SBP: 0 ± 3.92 DBP: 2.0 ± 3.31
Wu et al. [41]	China	F/M	48.2, 48.6	94	MetS	In diet	30 g/day	Lifestyle counselling AHA guidelines	12	Apo A: −0.07 ± 0.29 Apo B: −0.06 ± 0.22 SBP: −8.2 ± 12.11	Apo A: −0.09 ± 0.29 Apo B: −0.07 ± 0.22 SBP: −7.0 ± 12.43
Gullapalli et al. [42]	India	M	37, 37	37	Stag 1 hypertension	In diet	18% total energy	Local diet	6	SBP: −18.12, 4.38 DBP: −8.18 ± 2.4	SBP: −1 ± 4.42 DBP: 1.28 ± 2.3
Ma et al. [27]	USA	F/M	58, 58	12	DM	In diet	56 g/day shelled	Control diet	8	SBP: 4.0 ± 9.2 DBP: 1.6 ± 4.6	SBP: −4.9 ± 11.7 DBP: −2.5 ± 6.4
Rajaram et al. [24]	USA	F/M	No	25	Moderately hyperlipidemic	In diet	42.5 g/day	Rich fish diet	4	Apo A: −1 ± 0.08 Apo B: −0.09 ± 0.13	Apo A: 0 ± 0.08 Apo B: 0.04 ± 0.13
Spaccarotella et al. [26]	USA	M	45 to 75, 65.9	21	Men at risk for prostate cancer	In diet	75 g/day	American diet	8	SBP: −3.05 ± 12.73 DBP: −3.45 ± 6.96	SBP: −1.65 ± 12.73 DBP: −2.5 ± 6.96
Mukuddem‐Petersen et al. [36]	New Zealand	F/M	45, 45	21	Metabolic syndrome	In diet	20% total energy	Control diet	8	SBP: 2.0 ± 3.81 DBP: 0.4 ± 3.24	SBP: 2.0 ± 6.46 DBP: 0.4 ± 3.10
Ros et al. [30]	Spain	F/M	55, 55	12	Hypercholesterolemia patient	In diet	18% total energy	Mediterranean diet	4	Apo A: −4 ± 0.03 Apo B: −0.15 ± 0.11 SBP: −4 ± 7.6 DBP: −3.0 ± 4.02	Apo A: −2 ± 0.03 Apo B: −0.09 ± 0.10 SBP: −4 ± 7.4 DBP: −5.0 ± 4.35
Zambon et al. [28]	USA	F/M	56, 56	25	Hypercholesterolemia patient	In diet	18% total energy	Mediterranean diet	6	Apo A: −0.08 ± 0.1 Apo B: −0.2 ± 0.1	Apo A: −0.09 ± 0.1 Apo B: −0.13 ± 0.1
Chisholm et al. [35]	New Zealand	M	45, 46.8	16	Moderately hyperlipidemic	In diet	20% total energy	Low caloric diet	4	Apo A: 0.02 ± 0.11 Apo B: −0.13 ± 0.13	Apo A: 0.01 ± 0.10 Apo B: −0.08 ± 0.14

Abbreviations: CKD, Chronic kidney disease; CVD, Cardiovascular disease; F, Female; M, Male; MetS, Metabolic syndrome; PCOS, Polycystic ovary syndrome; T2DM, Diabetes mellitus Type 2.

### 3.3 Risk of Bias and Meta‐Evidence

The results of quality assessment based on Cochrane questionnaire for all included studies are presented in Table 2. Based on the GRADE approach, the quality of evidence was moderate for ApoB, low for ApoA1 and DBP, and very low for SBP (Table 3).

**TABLE 2:** Results of risk of bias assessment for randomised clinical trials included in the present study.

Author, year	Random sequence generation	Allocation concealment	Reporting bias	Other sources of bias	Performance bias	Detection bias	Attrition bias
Abdrabalnabi et al. [19]	U	L	U	L	L	L	L
Sanchis et al. [31]	L	L	U	L	L	L	L
Hwang et al. [37]	L	U	L	L	U	U	L
Fatahi et al. [33]	L	H	L	L	H	H	L
Rabiei et al. [32]	L	L	L	L	L	L	L
Ndanuko et al. [38]	L	L	L	L	L	L	L
Zibaeenezhad et al. [34]	L	L	L	L	L	L	L
Rock et al. [25]	L	L	L	L	L	L	L
Njike et al. [23]	L	U	L	L	U	U	L
Burns‐Whitmore et al. [20]	U	L	L	L	L	L	L
Lee et al. [39]	U	U	U	L	U	U	L
Wu et al. [11]	L	U	L	L	U	U	L
Olmedilla‐Alonso et al. [29]	U	H	U	L	H	H	L
Katz et al. [22]	U	L	L	L	L	L	L
Kalgaonkar et al. [21]	U	U	U	L	U	U	L
Din et al. [40]	L	L	U	L	L	L	L
Wu et al. [41]	U	U	L	L	U	U	L
Gullapalli et al. [42]	U	U	U	L	U	U	L
Ma et al. [27]	U	U	U	L	U	U	L
Rajaram et al. [24]	L	U	U	L	U	U	L
Spaccarotella et al. [26]	U	U	U	L	U	U	L
Mukuddem‐Petersen et al. [36]	L	U	U	L	U	U	L
Ros et al. [30]	U	U	U	L	U	U	L
Zambon et al. [28]	U	U	U	L	U	U	L
Chisholm et al. [35]	U	U	U	L	U	U	L

**TABLE 3:** Summary of findings and quality of evidence assessment using the GRADE approach.

	No of patients (meta‐analysis)	WMD (95% CI)	Risk of bias	Inconsistency	Indirectness	Imprecision	Publication bias	Quality of evidence
ApoA1	381 (6)	−0.50 (−1.34, 0.33)	Not serious	Not serious	Serious	Serious	Not serious	Low
ApoB	464 (8)	−0.06 (−0.10, −0.01)	Not serious	Not serious	Serious	Not serious	Not serious	Moderate
SBP	1950 (20)	−1.20 (−4.02, 1.61)	Not serious	Not serious	Serious	Serious	serious	Very Low
DBP	1864 (18)	−0.44 (−2.55, 1.67)	Not serious	Not serious	Serious	Serious	Not serious	Low

Abbreviations: ApoA1, Apolipoprotein A1; ApoB, Apolipoprotein B; DBP, Diastolic blood pressure; SBP, Systolic blood pressure.

### 3.4 Walnut Administration on ApoA1

**FIGURE 2:**
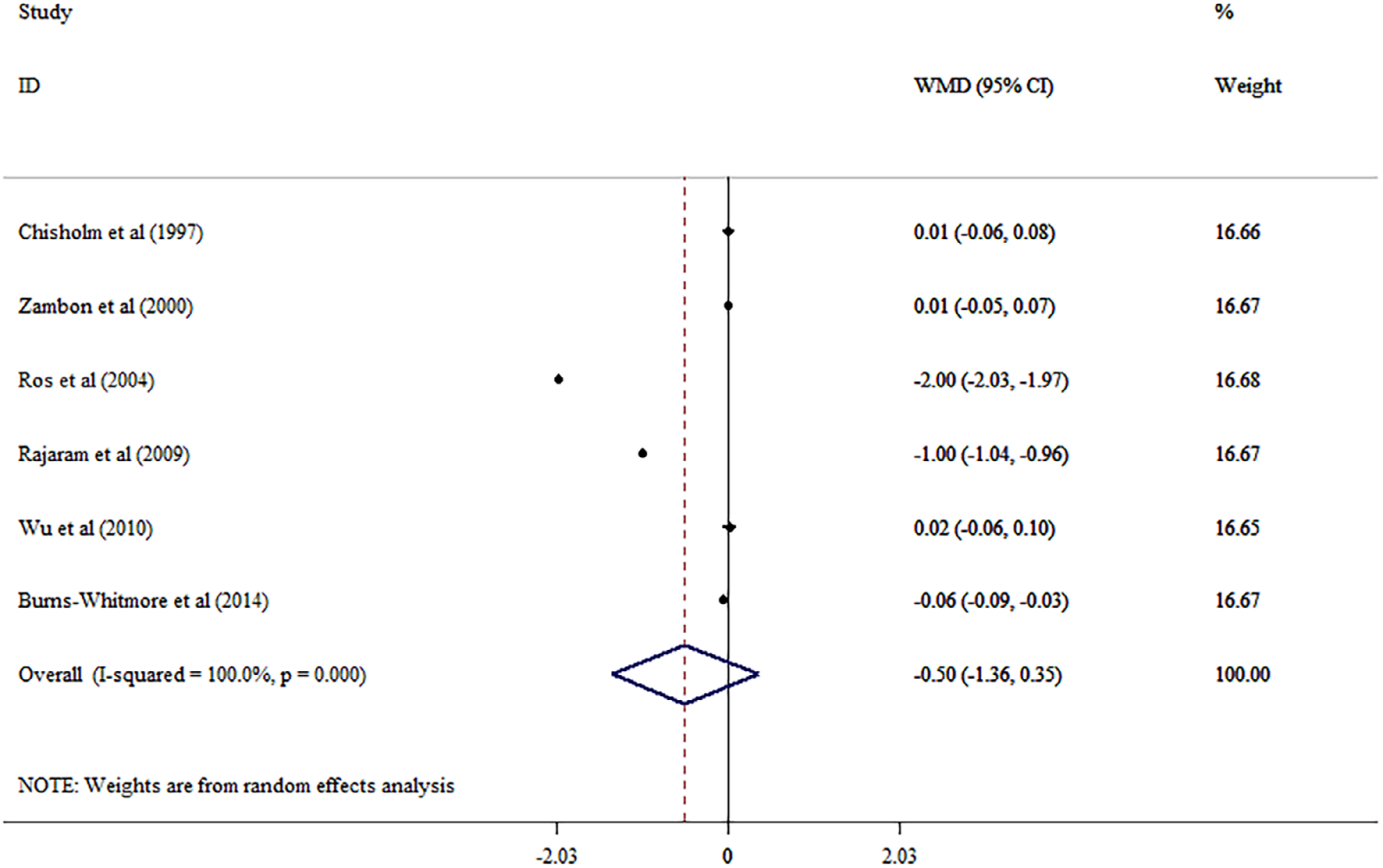
Forest plot detailing mean difference and 95% confidence intervals (CIs) the effects of walnut supplementation on Apo A levels.

Combining effect sizes from ten eligible studies with 381 participants showed no significant changes based on walnut‐enriched diet (WMD = −0.50; 95% CI: −1.34, 0.33, *p* = 0.249), with significant heterogeneity across the studies (*I* ^2^ = 100%, *p* < 0.001) (Figure 2). Subgroup analyses reported no significant effects for walnut on ApoA1 level after subgrouping by mean age, sample size, health condition and duration (Table 4). Also, no significant difference was seen by removing a single study using sensitivity analysis. No linear relationship was observed between effect size and age, intervention duration and dosage using meta regression analysis.

**TABLE 4:** Pooled estimate effects of walnut on apolipoproteins and blood pressure.

Group	No. of comparisons	WMD (95% CI)	*p*	*I* ^2^ (%)	*p*‐heterogeneity
Apo A1					
Age range (years old)					
< 45	3	−0.35 (−1.02, 0.31)	0.302	99.8	< 0.001
≥ 45	3	−0.66 (−2.22, 0.90)	0.409	100	< 0.001
Sample size					
< 45	3	−0.68 (−2.17, 0.80)	0.367	100	< 0.001
≥ 45	3	−0.32 (−1.06, 0.42)	0.392	99.8	< 0.001
Health condition					
Hyperlipidemic	4	−0.75 (−1.78, 0.29)	0.158	100	< 0.001
Metabolic syndrome	1	0.02 (−0.06, 0.10)	0.635	—	—
Healthy	1	−0.06 (−0.09, −0.03)	< 0.001	—	—
Duration (weeks)					
< 4	3	−1.00 (−2.06, 0.06)	0.065	99.9	< 0.001
≥ 4	3	−0.02 (−0.07, 0.04)	0.546	69.8	0.037
Apo B					
Age range (years old)					
< 45	4	−0.09 (−0.14, −0.03)	0.004	63.5	0.041
≥ 45	4	−0.03 (−0.07, 0.00)	0.061	16.6	0.308
Sample size					
< 45	4	−0.07 (−0.13, −0.01)	0.027	59.2	0.062
≥ 45	4	−0.05 (−0.11, 0.00)	0.058	66.9	0.028
Health condition					
Hyperlipidemic	4	−0.08 (−0.12, −0.04)	< 0.001	0	0.466
Metabolic syndrome	1	0.01 (−0.05, 0.07)	0.755	—	—
PCOS	1	−0.01 (−0.08, 0.06)	0.790	—	—
Healthy	2	−0.08 (−0.18, 0.02)	0.101	81.0	0.022
Duration (weeks)					
< 4	3	−0.09 (−0.14, −0.04)	0.001	13.3	0.316
≥ 4	5	−0.05 (−0.10, 0.00)	0.064	71.0	0.008
SBP					
Age range (years old)					
< 45	5	−2.62 (−11.87, 6.63)	0.579	97.7	< 0.001
≥ 45	15	−0.91 (−2.17, 0.35)	0.155	36.2	0.080
Sample size					
< 45	8	−0.24 (−3.08, 2.61)	0.871	59.9	0.015
≥ 45	12	−1.68 (−5.58, 2.22)	0.397	94.6	< 0.001
Health condition					
Overweight and obese	5	−0.63 (−2.69, 1.44)	0.551	49.3	0.096
Hypercholesterolemia	1	0.00 (−6.49, 6.49)	1.000	—	—
Metabolic syndrome	5	−0.30 (−1.68, 1.08)	0.669	0	0.589
Stage 1 hypertension	1	−17.12 (−19.30, −14.94)	< 0.001	—	—
DM	4	−0.89 (−4.44, 2.66)	0.624	68.4	0.023
CKD	2	0.85 (−16.16, 17.87)	0.922	56.8	0.128
Men at risk for prostate cancer	1	−1.40 (−9.11, 6.31)	0.722	—	—
Healthy	1	3.00 (0.03, 5.97)	0.048	—	—
Duration (weeks)					
≤ 6	6	−2.61 (−12.83, 7.62)	0.617	96.4	< 0.001
> 6	14	−0.69 (−1.87, 0.50)	0.257	41.2	0.053
DBP					
Age range (years old)					
< 45	5	−1.04 (−7.35, 5.27)	0.747	93.3	< 0.001
≥ 45	13	0.01 (−0.82, 0.85)	0.974	33.1	0.117
Sample size					
< 45	8	0.03 (−1.54, 1.59)	0.975	56.7	0.024
≥ 45	10	−0.76 (−4.01, 2.50)	0.648	95.9	< 0.001
Health condition					
Overweight and obese	5	−0.08 (−1.29, 1.14)	0.900	0	0.752
Hypercholesterolemia	1	2.00 (−1.64, 5.64)	0.282	—	—
Metabolic syndrome	4	1.10 (−0.48, 2.67)	0.172	65.6	0.033
Stage 1 hypertension	1	−9.46 (−10.62, −8.30)	< 0.001	—	—
DM	4	−0.26 (−3.06, 2.54)	0.858	78.7	0.003
CKD	1	−2.00 (−9.17, 5.17)	0.584	—	—
Men at risk for prostate cancer	1	−0.95 (−5.16, 3.26)	0.659	—	—
Healthy	1	1.00 (−1.44, 3.44)	0.421	—	—
Duration (weeks)					
≤ 6	4	−2.21 (−9.39, 4.98)	0.548	96.4	< 0.001
> 6	14	0.23 (−0.71, 1.17)	0.634	53.0	0.010

### 3.5 Walnut Administration on ApoB

**FIGURE 3:**
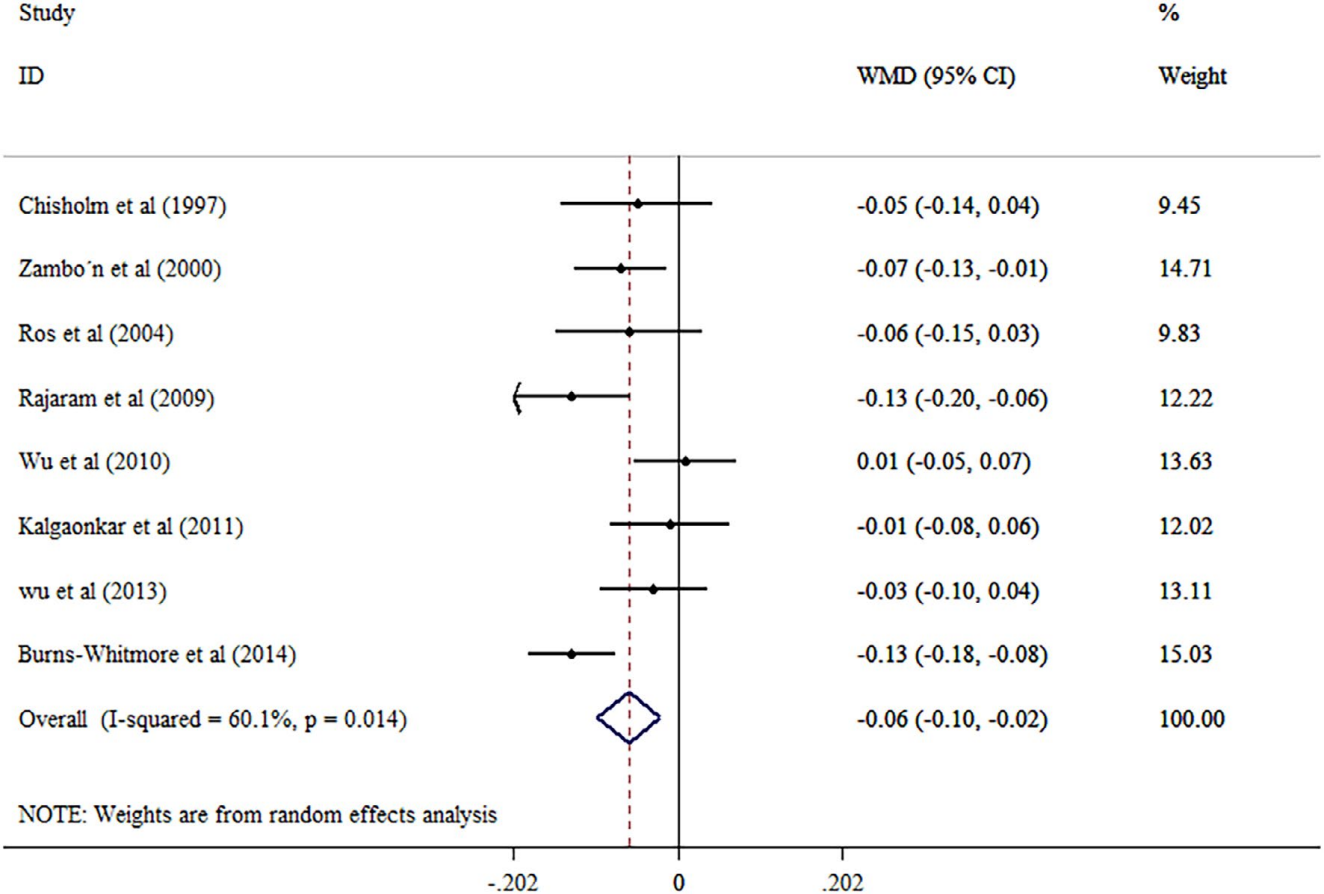
Forest plot detailing mean difference and 95% confidence intervals (CIs) for the effects of walnut supplementation on ApoB levels.

The pooled outcomes from the random‐effects model of eight studies (464 participants) revealed that walnut consumption reduces the ApoB level in a significant manner (WMD = −0.06; 95% CI: −0.10, −0.01, *p* = 0.002) with a considerable between‐study heterogeneity (*I* ^2^ = 74.6%, *p* < 0.001) (Figure 3). Moreover, subgroup analysis based on age (> 45 years old: *I* ^2^ = 16.6%, *p* = 0.308), health condition (hyperlipidemic: *I* ^2^ = 0.0%, *p* = 0.466), and treatment duration (< 4 weeks: *I* ^2^ = 13.3%, *p* = 0.316) disappeared the heterogeneity. It has been shown that younger adults (< 45 years old) (*p* = 0.027) and hyperlipidemic individuals (*p* < 0.001) benefit more from adherence to walnut intake, exhibiting a significant reduction in ApoB levels. Furthermore, short‐term treatment (≤ 4 weeks) proved to be more effective in lowering ApoB levels (*p* = 0.001) (Table 4). Sensitivity analyses illustrated that the overall WMD of ApoB does not depend on any single study effect.

### 3.6 Walnut Administration on SBP

**FIGURE 4:**
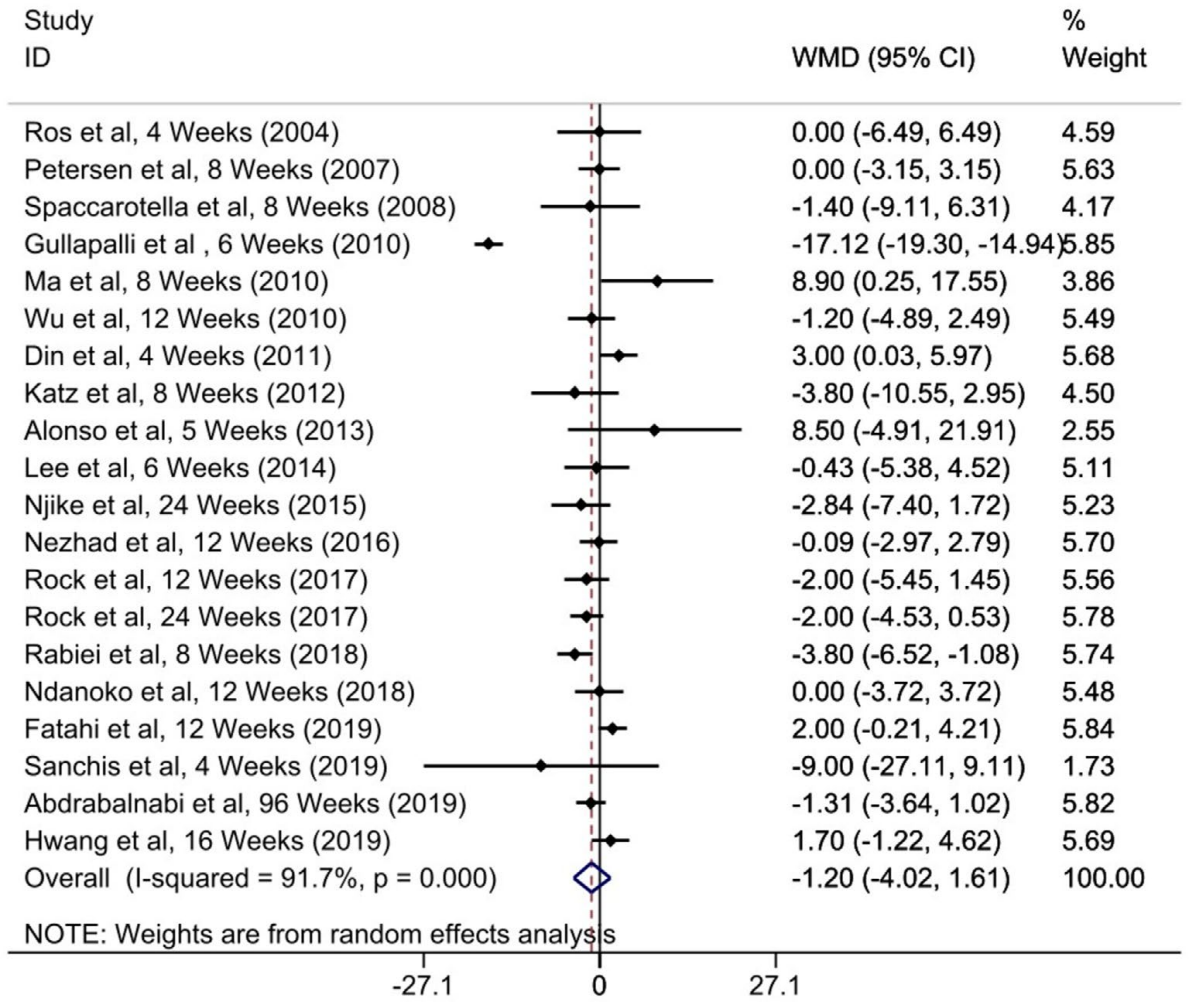
Forest plot detailing mean difference and 95% confidence intervals (CIs) the effects of walnut supplementation on SBP levels.

Pooling effect sizes from 19 publications comprising 20 treatment arms containing 1950 participants demonstrated a non‐significant effect for walnut (WMD = −1.20; 95% CI: −4.02, 1.61, *p* = 0.401; *I* ^2^ = 91.7%, *p*‐heterogeneity < 0.001) (Figure 4). This considerable between‐study heterogeneity was reduced using subgroup analysis by participant's age (*I* ^2^ = 36.2%, *p* = 0.080), treatment duration (*I* ^2^ = 41.2%, *p* = 0.053), and health condition (*I* ^2^ = 0.0%, *p* = 0.589) (Table 4). There was no significant difference in removing a single study using sensitivity analysis. No linear relationship was observed between effect size and age, intervention duration, and dosage using meta‐regression analysis. Visual inspection of the funnel plot (Figure S1) revealed an asymmetry, and Egger's and Begg's tests were *p* = 0.331 and 0.559, respectively. Accordingly, the trim‐and‐fill method was applied to simulate a model with no publication bias. Accordingly, trim and fill analysis was performed with 6 imputations (26 studies) (WMD = −2.99 mmHg; 95% CI: −5.42, −0.559, *p* = 0.016) (Figure S2). Given the exploratory nature of the trim‐and‐fill method and the presence of heterogeneity, these findings should be interpreted cautiously and considered hypothesis‐generating rather than confirmatory.

### 3.7 Walnut Administration on DBP

**FIGURE 5:**
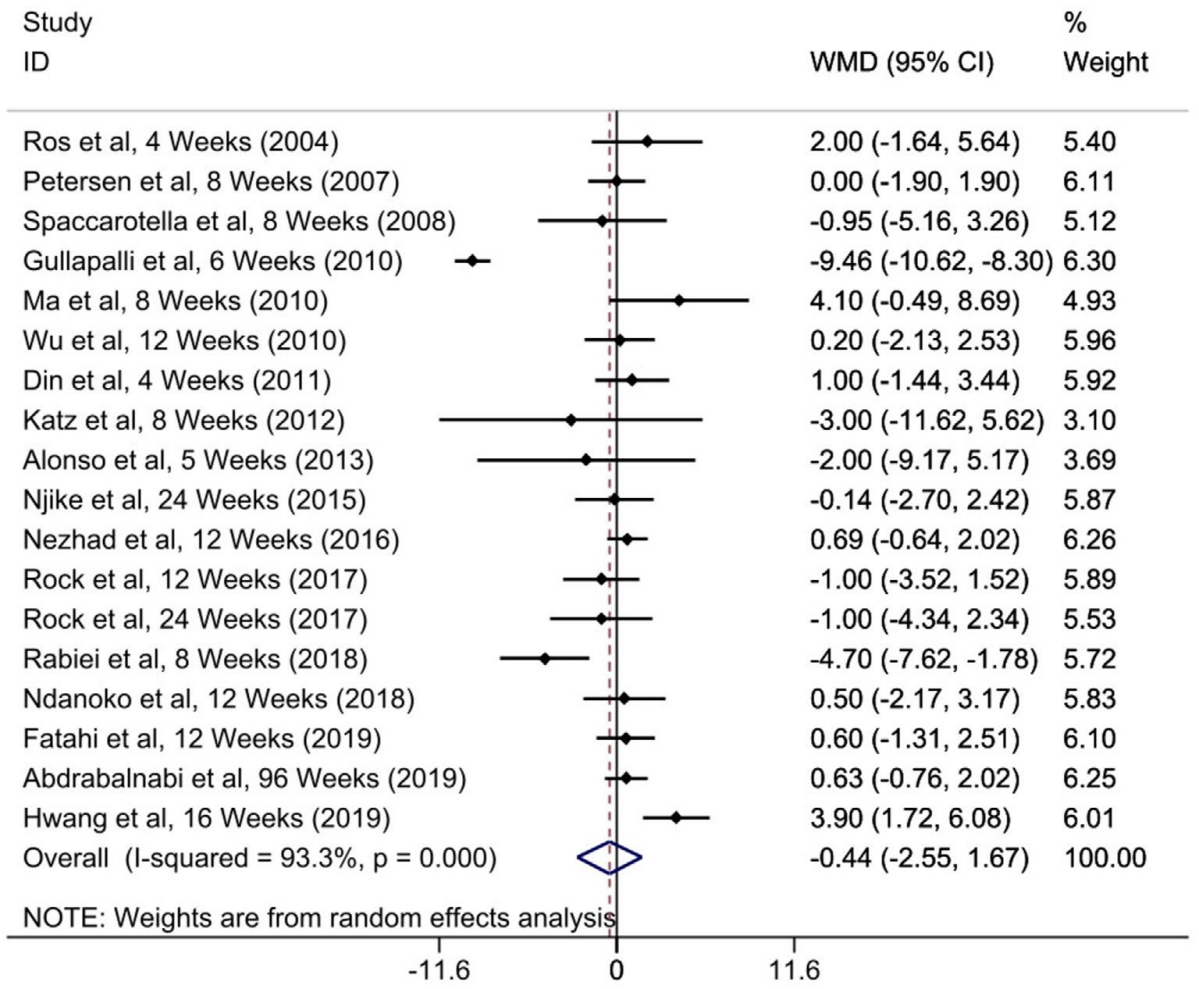
Forest plot detailing mean difference and 95% confidence intervals (CIs) of the effects of walnut supplementation on DBP levels.

Eighteen studies with 1864 individuals (case = 954, control = 910) investigated the effect of walnut on DBP as a result measure. The combined outcome from the random‐effects model specified that walnut treatment did not alter SBP significantly (WMD = −0.44; 95% CI: −2.55, 1.67, *p* = 0.682; *I* ^2^ = 93.3%, *p* < 0.001) (Figure 5). Subgroup analysis indicated that there was no significant effect of walnut on DBP after subgroup analysis by mean age, sample size, health condition and duration of treatment (Table 4). There was no significant difference in removing a single study using sensitivity analysis. No linear relationship was observed between effect size and age, intervention duration and dosage using meta regression analysis. No small‐study effects were seen performing Egger's and Begg's tests (*p* = 0.188 and 0.053, respectively). However, funnel plot (Figure S3) showed an asymmetric distribution of publication around the WMD. Then, trim and fill approach was performed with a new effect size with inserting 5 fictitious publications (WMD = −1.51 mmHg; 95% CI: −3.26, 0.23; *p* = 0.089) (Figure S4).

## 4 Discussion

The current review summarised the results of 2155 individuals who incorporated walnuts into their diet. We included estimates for the effect of a walnut‐enriched diet in comparison to the control group in terms of apolipoproteins and BP. Accordingly, our findings indicate that walnut consumption did not significantly affect ApoA1, SBP, and DBP levels compared to the control group. However, a walnut‐enriched diet was associated with a significant reduction in ApoB levels among adults, suggesting a potential cardiovascular benefit.

Evidence suggests that walnuts are a nutrient‐dense food rich in monounsaturated fatty acids (MUFA) and polyunsaturated fatty acids (PUFA) [43, 44]. They are particularly high in LA and ALA [45]. Research indicates that ALA plays a crucial role in improving lipid metabolism, contributing to a reduction in lipid concentrations [44]. Indeed, these plant‐based fatty acids available in walnut interfere with cholesterol absorption and promote receptor‐mediated LDL‐c clearance [45]. Subsequently, ApoB, as a primary apolipoprotein which carries chylomicrons, low‐density lipoprotein (LDL), very low‐density lipoprotein (VLDL), intermediate‐density lipoprotein (IDL), and lipoprotein (a), may result in declined ApoB level [46]. Also, it seems that compounds found in walnuts limit triglyceride (TG) availability for VLDL assembly, thereby inhibiting ApoB secretion [46]. In this regard, Banel et al. point out the lipid‐lowering effects of walnuts which could decrease the total cholesterol (TC), LDL‐c, and TG levels. While it was not successful in affecting HDL‐c levels [47]. Consistent with earlier meta‐analyses, Guasch‐Ferré et al. reported a decrease in TC, LDL‐c, TG, and ApoB concentrations following adherence to a walnut‐enriched diet [48]. Similarly, another meta‐analysis reported that walnuts significantly lowered TC, LDL‐c, and Apo A and B levels [49]. Also, atherogenic lipoproteins trigger endothelial cells to initiate inflammatory cascades [50, 51]. ApoB, as a component of atherogenic lipoproteins, is significantly reduced with high adherence to a walnut‐enriched diet, which is associated with a decreased risk of cardiometabolic diseases [27, 52, 53]. Beyond the biochemical mechanisms, the real‐world applicability of walnut consumption should be considered. Although walnuts are nutrient‐dense and widely available, their cost, energy density, and long‐term adherence may limit sustained intake in some populations. Moreover, the benefits are likely influenced by dietary substitution effects, with greater improvements when walnuts replace less healthful foods rather than being added on top of habitual diets. Therefore, walnut consumption may be most effective as part of an overall heart‐healthy dietary pattern rather than as a standalone intervention.

However, subgroup analysis revealed that a walnut‐enriched diet can have more pronounced effects on ApoB in hyperlipidemic patients. Since hyperlipidemic patients present higher levels of TG leading to increased VLDL particles which contain ApoB [54]. Likewise, walnut intervention seems to be more effective in hyperlipidemic patients with elevated lipid levels [54]. Also, a pooled analysis of 25 clinical trials demonstrated that incorporating nuts into the diet leads to decreased TC, LDL‐c, and LDL/HDL ratio in normal and hypercholesterolemic patients [55]. Also, our data analyses indicated age‐specific effects for walnut in terms of ApoB, which shows that walnut intake in younger adults (< 45 years old) could reduce ApoB levels significantly. Whereas, Guasch‐Ferré et al. illustrated that walnut intake led to significant reductions in ApoB levels among healthy adults [48]. It may be attributed to better metabolic function compared to older individuals which could enhance their response to dietary interventions. Moreover, short‐term consumption of walnut was accompanied by a significant reduction in ApoB levels in the present study too. The probable mechanism may be attributed to the metabolic adaptation phenomenon. It seems that metabolic adaptation induced by long‐term adherence to dietary interventions may affect the magnitude of the body's response. Furthermore, no significant effects of walnuts were seen on blood pressure compared to the control group. In line with our findings, several meta‐analyses reported no significant changes in blood pressure based on total nuts and walnuts consumption [48, 49, 56]. Furthermore, potential publication bias was observed in blood pressure outcomes. To address this, the trim‐and‐fill approach was applied to reach a more robust and unbiased estimation of the pooled effect sizes. This issue reinforces the validity of our findings and underscores the potential benefit of walnut consumption on the regulation of blood pressure.

Considerable variability in the ApoA1, SBP and DBP results was observed (*I* ^2^ > 90) indicating significant variability between studies. This variability may be due to differences in the characteristics of the participants (e.g., age, medical condition, baseline lipid profile), the background diet, the dose and form of the nuts (whole, sliced or oil), the duration of the intervention and the overall quality of the study. Although subgroup and sensitivity analyses have been performed, there is still considerable residual variability which highlights the need for careful interpretation of pooled estimates. Future well‐designed studies with standardised interventions and more homogeneous populations are justified to further elucidate these effects.

Although walnut consumption was associated with a statistically significant reduction in ApoB, the absolute magnitude of the effect was modest (WMD = 0.06 g per litre). This reduction is smaller than that usually obtained with statin treatment, but comparable to the effects seen with other single dietary interventions and with nut consumption in general. The observed benefit may therefore be clinically relevant as part of a general heart healthy diet, rather than as a stand‐alone strategy. In addition, the presence of moderate variability between studies suggests that individual responses may differ depending on the design of the studies, population characteristics and dietary background.

There are some potential limitations to this review and meta‐analysis which should be considered. First, although participants showed high compliance with walnut‐enriched diet. However, variation in compliance may lead to underestimation of outcomes. Second, a lower number of included studies and smaller sample size affect the statistical power of the study. Thirdly, a smaller number of the studies included were double‐blind studies, which may minimise bias. Many of the included studies had unclear or high risk of bias, especially with regard to performance and detection bias, which may limit the reliability of the pooled estimates and justify a cautious interpretation of the findings.

## 5 Conclusion

This updated meta‐analysis provides a comprehensive approach in relation to walnut consumption and its effect on apolipoproteins and blood pressure as cardiovascular risk factors. The present meta‐analysis of controlled trials provides robust evidence for the reducing effects of walnut intake on ApoB level. However, no significant changes were shown in terms of SBP and DBP following a walnut‐enriched diet among adults.

## Author Contributions

V.M. and M.M. contributed in the systematic search and data extraction. S.M.H.‐R. and M.F. contributed in the statistical analyses and data interpretation. V.M. and F.S.H. contributes in manuscript drafting and data interpretation. F.S. and M.F. critically evaluated the analysis and edited the M.S. All authors approved the final manuscript for submission.

## Funding

The authors have nothing to report.

## Ethics Statement

The authors have nothing to report.

## Consent

The authors have nothing to report.

## Conflicts of Interest

The authors declare no conflicts of interest.

## Supporting information


**Appendix S1:** edm270171‐sup‐0001‐AppendixS1.docx.

## Data Availability

The data that support the findings of this study are available from the corresponding author upon reasonable request.
