# Supplementary material for: Does Walnut Supplementation Have Favourable Effect Apolipoprotein A, B and Blood Pressure? A Systematic Review, Meta‐Analysis and Meta‐Evidence of Randomised Clinical Trials
Source: Endocrinol Diabetes Metab. 2026 Mar 20;9(2):e70171. doi: 10.1002/edm2.70171 (PMC13093844; doi:10.1002/edm2.70171)
Supplement: Supplementary file 1 — Appendix S1: edm270171‐sup‐0001‐AppendixS1.docx. [file EDM2-9-e70171-s001.docx]

(“walnut” OR “walnuts” OR “juglans”) AND (“blood pressure” OR “systolic blood pressure” OR “diastolic blood pres- sure” OR “SBP” OR “DBP” OR “hypertension” OR "Apolipoproteins" OR "Apolipoprotein B’ OR "Apolipoprotein A-I" OR "Apolipoprotein B-100 OR "Apolipoprotein A” OR "Apo A" OR "Apo B" ) AND (“clinical trials” OR “clinical trial” OR “cross-over studies” OR “double-blind method” OR “single-blind method” OR “random allocation” OR “RCT” OR “random” OR “randomly” OR “placebo” OR “assignment” OR “intervention stud- ies” OR “intervention” OR “controlled trial” OR “randomized” OR “Trial”OR “randomised”).

**Supplementary Table1.** Pattern of Search


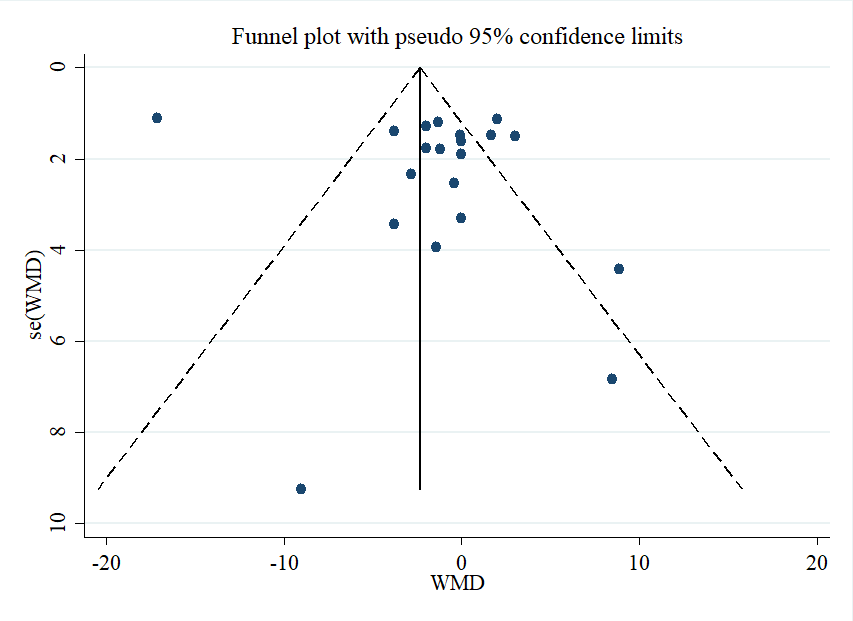
**Supplementary Figure1.** Funnel plot for SBP.


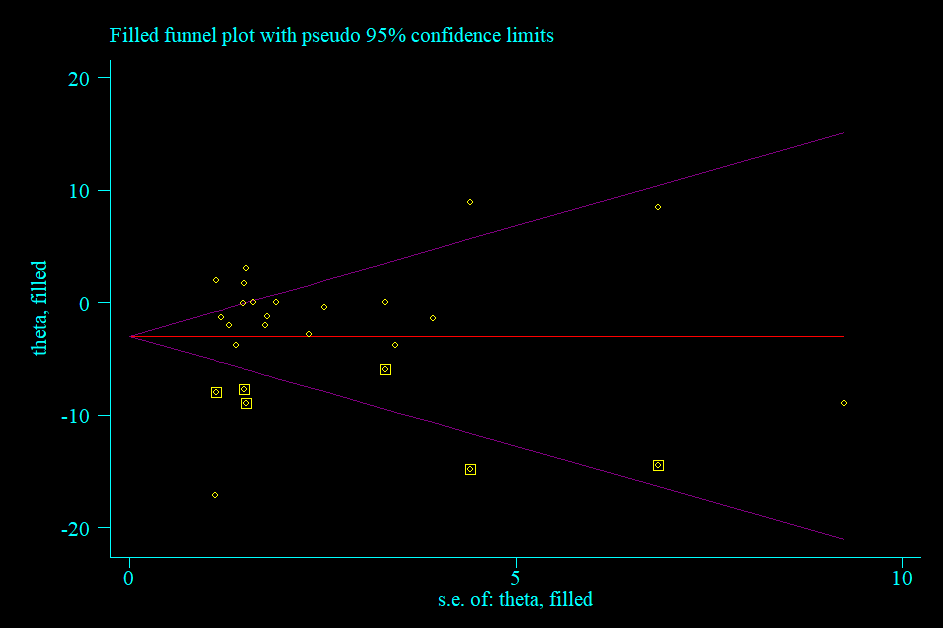


**Supplementary Figure2.** Trim and filled for SBP.


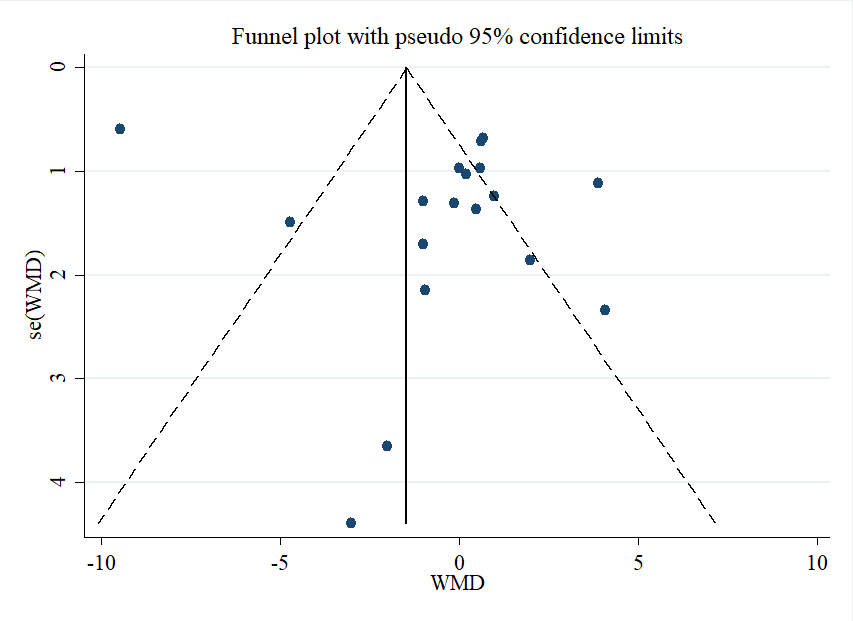


**Supplementary Figure3.** Funnel plot for DBP


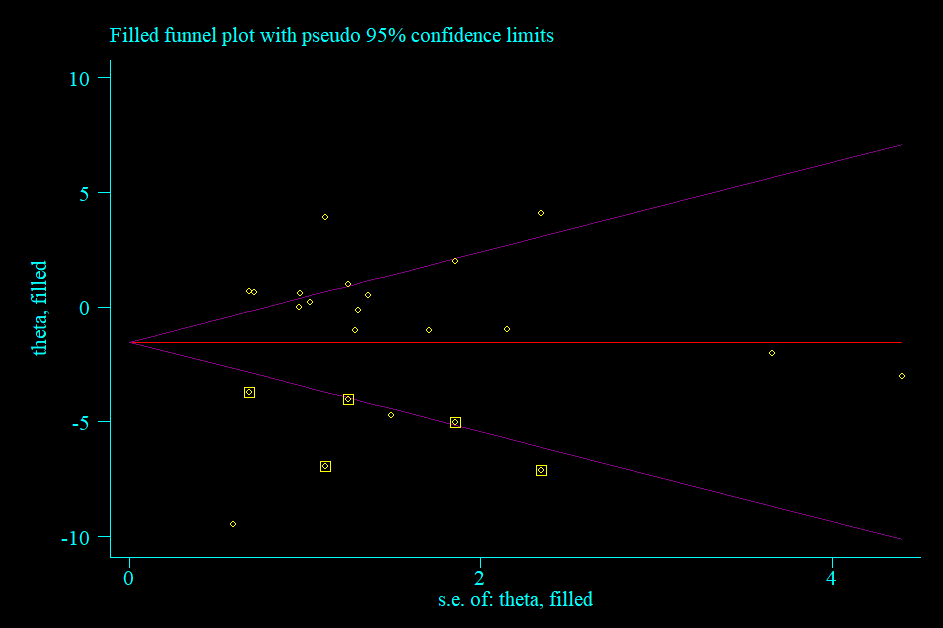


**Supplementary Figure4.** Trim and filled for DBP.

| **Supplementary Table 2.** A summary of excluded articles after full text review | |
| --- | --- |
| **Author, Year (Ref.)** | **Reason** |
| Canales, 2014 (Canales et al., 2007) | Mismatch with Review Criteria |
| Carol E., 2015 (O’Neil, Fulgoni III, & Nicklas, 2015) | Mismatch with Review Criteria |
| Iwamoto, 1995 (Iwamoto et al., 2000) | Retracted |
| Pan, 2013 (Pan, Sun, Manson, Willett, & Hu, 2013) | Mismatch with Review Criteria |
| Tindall, 2019 (Tindall et al., 2019) | Mismatch with Review Criteria |
| Torabian, 2010 (Torabian et al., 2010) | Mismatch with Review Criteria |
| KAMRAN TAJ, 2010 (Taj, Cheema, & Hassani, 2010) | Mismatch with Review Criteria |
| Sabate,1993 (Sabaté et al., 1993) | Mismatch with Review Criteria |

Canales, A., Benedi, J., Nus, M., Librelotto, J., Sánchez-Montero, J. M., & Sánchez-Muniz, F. J. (2007). Effect of walnut-enriched restructured meat in the antioxidant status of overweight/obese senior subjects with at least one extra CHD-risk factor. *Journal of the American College of Nutrition, 26*(3), 225-232.

Iwamoto, M., Sato, M., Kono, M., Hirooka, Y., Sakai, K., Takeshita, A., & Imaizumi, K. (2000). Retracted: Walnuts Lower Serum Cholesterol in Japanese Men and Women. *The Journal of nutrition, 130*(2), 171-176.

O’Neil, C. E., Fulgoni III, V. L., & Nicklas, T. A. (2015). Tree Nut consumption is associated with better adiposity measures and cardiovascular and metabolic syndrome health risk factors in US Adults: NHANES 2005–2010. *Nutrition journal, 14*(1), 64.

Pan, A., Sun, Q., Manson, J. E., Willett, W. C., & Hu, F. B. (2013). Walnut consumption is associated with lower risk of type 2 diabetes in women. *The Journal of nutrition, 143*(4), 512-518.

Sabaté, J., Fraser, G. E., Burke, K., Knutsen, S. F., Bennett, H., & Lindsted, K. D. (1993). Effects of walnuts on serum lipid levels and blood pressure in normal men. *N Engl J Med, 328*(9), 603-607. doi: 10.1056/nejm199303043280902

Taj, M. K., Cheema, A. M., & Hassani, I. (2010). Lipid Profile in Supplementation Walnut and Almond in Hypertension Subjects. *Pakistan Journal of Medical & Health Sciences, 4*(3), 172-174.

Tindall, A. M., Petersen, K. S., Skulas‐Ray, A. C., Richter, C. K., Proctor, D. N., & Kris‐Etherton, P. M. (2019). Replacing saturated fat with walnuts or vegetable oils improves central blood pressure and serum lipids in adults at risk for cardiovascular disease: a randomized controlled‐feeding trial. *Journal of the American Heart Association, 8*(9), e011512.

Torabian, S., Haddad, E., Cordero-MacIntyre, Z., Tanzman, J., Fernandez, M., & Sabate, J. (2010). Long-term walnut supplementation without dietary advice induces favorable serum lipid changes in free-living individuals. *European journal of clinical nutrition, 64*(3), 274-279.
